# Calorie Labeling in a Rural Middle School Influences Food Selection: Findings from Community-Based Participatory Research

**DOI:** 10.1155/2015/531690

**Published:** 2015-03-22

**Authors:** Monica Hunsberger, Paul McGinnis, Jamie Smith, Beth Ann Beamer, Jean O'Malley

**Affiliations:** ^1^University of Gothenburg, Public Health Epidemiology and Community Medicine, P.O. Box 454, 405 30 Gothenburg, Sweden; ^2^Eastern Oregon Coordinated Care Organization, 309 E 2nd Street, The Dalles, OR 97058, USA; ^3^Jefferson County School District 509-J, 445 SE Buff Street, Madras, OR 97741, USA; ^4^St. Charles Health System, 470 NE A Street Madras, OR 97741, USA; ^5^Oregon Clinical & Translational Research Institute, Oregon Health & Science University, Portland, OR 97239, USA

## Abstract

*Background.* Calorie labeling at the point-of-purchase in chain restaurants has been shown to reduce energy intake.* Objective.* To investigate the impact of point-of-purchase calorie information at one rural middle school.* Methods.* With a community-based participatory research framework a mixed method approach was used to evaluate the impact of point-of-purchase calorie information. Students in grades 6–8, dining at the school cafeteria January and February 2010, participated for 17 school days each month; in January a menu was offered in the usual manner without calorie labels; the same menu was prepared in February with the addition of calorie labels at point-of-purchase. Gross calories served per student were measured each day allowing for matched comparison by menu. In March/April of 2010, 32 students who ate in the cafeteria 3 or more times per week were interviewed regarding their views on menu labeling.* Results.* Calorie consumption decreased by an average of 47 calories/day; fat intake reduced by 2.1 grams/day. Five main themes were consistent throughout the interviews.* Conclusion. *Point-of-purchase calorie labels can play a role in reducing the number of calories consumed by middle school age children at the lunch. The majority of students interviewed found the calorie labels helped them choose healthier food.

## 1. Introduction

Escalating rates of obesity may cause the current generation of children to have a life expectancy shorter than that of their parents [1]. Overweight and obese adolescents are now presenting with type 2 diabetes, dyslipidemia, and hypertension, diseases not previously seen in this age group [2, 3]. Swift action is needed to reverse these trends.

Schools are in a unique position to influence the diets of children and adolescents. Most students attend school for six or more hours per day, 180 days per year [4]. The majority of students consume a school lunch and some may also have a school breakfast, allowing over 47% of their total daily energy intake to be obtained in the school setting [5].

Menu labeling in chain and fast-food restaurants has received increasing attention as a policy to reduce energy intake. Findings show patrons making healthier, lower calorie food choices when calorie information is available [6, 7]. Encouraging findings at the local and state level have led to national legislation requiring menu labeling [8, 9]. Despite these positive results, a paucity of research exists on both the effect of menu labeling or calorie labeling in the school setting and on adolescents' opinions of labeling measures.

Jefferson County Middle School (JCMS) provides the ideal setting to examine the impact of menu labeling in schools. It is a public school situated in a low income area with just over 79% of its students entitled to free or reduced price cafeteria lunches and 64.6% of students are members of an ethnic minority. Although obesity is a national and international public health concern, research suggests that children and adolescents who live in multiethnic, low-income neighborhoods are at particularly high risk for obesity [10]. Adolescent obesity is a considerable problem at JCMS where 32.5% of sixth and eighth grade students were found to have a BMI above the 95th percentile, placing them in the obese category. Therefore, the Mountain View Community Health Improvement and Research Partnership, a community-based participatory research (CBPR) partnership, selected this school-based approach as one method aimed at the prevention of overweight.

To our knowledge only one other study has evaluated the impact of menu labeling in schools with college freshman and the results showed a positive impact [11]. We hypothesized calorie information at the point-of-purchase would influence students' food choices. The aim of the study was to investigate the impact of calorie posting at the point-of-purchase in a middle school cafeteria.

## 2. Methods

This study employed a mixed-method design including both qualitative and quantitative data collection. Ethical approval for the study was obtained by the Oregon Health and Science University and the North West Indian Health Region. The formation of the Mountain View Community Health Improvement and Research Partnership has been described in detail previously [12]. In brief, this partnership includes a wide range of stakeholders with a common goal of improving community health.

### 2.1. Subject Recruitment and Setting

The study was carried out in the Jefferson County Middle School (JCMS), Madras, Oregon, United States. The school is situated in a low-income community and participates in the United States Department of Agriculture (USDA) National School Lunch Program. Students included males and females aged 11 to 15 years of age in 6th–8th grade. On average, seventy-eight percent (daily average *N* = 531) of JCMS students participated in the school lunch program over the two-month study period and were included in the analysis of gross calories purchased per student. In January 2010 the cafeteria menu was presented as “usual” without calorie labels. In February 2010 the identical menu was presented with calorie labels at the point-of-purchase. Calorie labels were printed, laminated, and placed above the corresponding items on the sneeze guard for both the hot and cold food lines. Gross calorie consumption was calculated by weighing each food and beverage option before service and after service, imputing weights into a USDA approved nutrient database (Nutrikids), and dividing by the total number of children served per day. Gross calorie consumption assesses changes in total calories on a group level rather than on an individual level. Faculty and staff were excluded and used a separate salad bar for the duration of the study to uphold the accuracy of the weights. During the study period, after school sport offerings remain identical, girls can choose to play basketball and boys wrestling.

To recruit participants for the qualitative interviews, a letter of invitation was sent to students that ate lunch three or more times per week in all three grades. Consent for the parents and assent for the minor age participants were included along with a postage paid return envelope. Students also had the option to directly return the forms to the lunch room leader who was part of the CBPR partnership. To encourage participation, a gift card valued at $10.00 for either a local grocery store or subway sandwich shop was offered to the students. All interviews were conducted at JCMS during the school day.

Interviews followed a guided approach to ensure that the same general areas were discussed with each interviewee [13]. Interviews were audiotaped, transcribed verbatim, and analyzed for key themes by three researchers. First the transcripts were reviewed to achieve familiarity with the data. Key statements were coded deductively with codes conforming to interview guide questions. Next, the transcripts were reread to inductively identify areas not detected with the “top down” method of analysis and then recoded to incorporate the emergent codes [13]. Each code was reviewed and the five most relevant themes were identified.

### 2.2. Statistical Analysis

Statistical analysis was done using SAS 9.2. Consumption of menu items prepared on site was estimated by the difference between starting and ending weights; the nutrient composition of these items was estimated from the item recipes. Consumption of prepackaged items (e.g., milk, commercial salads) was estimated by the difference in units; nutrient composition for these items was estimated by the item labels. Per student fat and calorie and fat consumptions were computed and paired by menu matching. Pre- and postlabeling per student consumption of kilocalories fat and was analyzed using paired* t*-tests.

## 3. Results

As summarized in Table 1, the presence of calorie labels resulted in a mean decrease of 47 calories per student (95% CI = −77 to −18, *P* = 0.0040) and 2.1 grams of total fat per student (95% CI = −3.3 to −0.9, *P* = 0.0025).

After assessment of gross caloric changes, 32 students were interviewed. A summary of participants is shown in Table 2.

Following these 32 interviews, the qualitative analysis produced five significant themes. For each theme, the main points are presented along with relevant quotations (Table 3).

### 3.1. Obesity Epidemic and School Responsibility

Many students were aware of the adolescent obesity epidemic. One interviewee stated “kids are getting bigger and heavier nowadays than they used to be.” The students believed it was the schools responsibility to help stop this trend and aid the students in achieving a healthy weight. Many students were also eager to express their specific ideas how school could make the suggested changes. For example, students suggested announcing the healthier cafeteria options every morning to make choosing healthy food easier.

### 3.2. Nutritional Knowledge Was Related to Home Environment

Students were asked the extent to which their families discussed nutrition and used nutrition labels. Students' responses suggest that their knowledge of healthy eating is highly dependent on the nutrition practices of their parents and siblings. They tended to mimic the behavior of their family members when it came to reading labels stating “My mom, my sisters, and me, we look at the nutrition labels.” A lack of importance placed on nutrition in the home seemed to reinforce students' negative attitudes towards healthy eating and act as a further deterrent to menu label usage. Some parents had attempted to scare their children into consuming less. “My mom tries to scare me about calories; that way I won't have as much in a day.” However, these strategies often resulted in students having inaccurate nutrition knowledge. Overall, it was mainly students whose parents demonstrated an interest in nutrition that were positive about seeking nutritional information.

### 3.3. Taste Drives Intake

Most participants agreed that nutrition and being a healthy weight were important to them but taste was declared the most important factor. The appearance and nutritional content of food also rated highly. One student remarked that the only time he did not use the calorie labels to make a lower calorie choice was a when a certain food “looked really good.” Although many students considered nutrition information to be important, and widespread poor nutritional knowledge was evident. Very few students could correctly identify their energy requirements or understand the role of calories in achieving energy balance: “They're {calories} something that builds up inside your intestine.” This is despite studying nutrition in the mandatory health class.

### 3.4. Calorie Labels and Health

Most students considered displaying the calorie amounts to be important and one student expressed a desire for restaurants to also have the calorie counts on display. Although many of the interviewees had an awareness of the connection between obesity and chronic disease, a small number believed maintaining a healthy weight was primarily associated with image. When asked was nutrition information important to maintain a healthy weight, one student replied that he did not “judge people by their looks.” Furthermore, some students admitted having no interest in nutrition “I saw the calories but I didn't use them.”

Some students stated the calorie labels “helped {them} make healthier choices.” A positive side effect was that by choosing lower calorie items many students automatically chose lower fat foods “I switched from a side of chips to salad.” Many students were surprised by the high number of calories in some foods which lead them to choosing a lower calorie alternative “I was surprised how high the calories were so I took less.” In some cases the adolescents found that the labels did not impact the specific food they ate but the quantity, with many opting for smaller portions.

### 3.5. Not Hall Talk

When it came to the labels themselves, the color, font, size, and positioning of the labels were the main areas highlighted by the interviewees. As one student remarked “nobody would really have time just to stop right there and look at it because you have to keep on going.” Another student mentioned that “when we see it big, we want to know what that is, because you notice it more.” Students who did not use the labels did not mind their presence. “Some of my friends thought it was cool to have them and some of them didn't really care.” Overall, most students revealed that they would like to see the calorie information displayed but that it is only useful “if people actually read it, if they don't it's a waste of time.” However, most of the students interviewed stated they did not discuss the calorie labels with their friends.

## 4. Discussion

This mixed-method study examined both the change in gross calories consumed and students' opinions. Quantitative results demonstrated that calorie labels at the point-of-purchase decreased caloric consumption. Although some students claimed not to notice and/or use the calorie information during the interviews, they may simply be unaware of the impact calorie labels had on their selection of food or it may be due to reluctance among some students to admit to using the calorie labels. The later reason may be a more plausible explanation as the majority of interviewees also claimed not to have discussed nutrition in general with their peers.

Students in this study believed the school is responsible for helping them achieve and maintain a healthy weight. Students wanted healthier cafeteria choices along with nutrition information to guide their food decisions and facilitate healthier eating. To demonstrate their interest, students identified a number of potential healthy eating initiatives including providing “a better variety of fruit and vegetables,” “more selections of healthy food,” “food with less fat,” and “with lower calories.”

Students also recognized that by introducing menu labels the school was trying to take measures to combat obesity, “with labels they are {school} at least attempting to do something about it.” Further, this study demonstrated that students whose parents discussed nutrition at home and used nutritional labels were more likely to (1) notice and use the calorie labels, (2) use nutritional labels on prepackaged food, (3) have a greater overall awareness about nutrition and healthy eating, and (4) place a greater importance on nutrition in general. The third theme indicated that students chose products from the cafeteria based on taste, nutrition, and food appearance. Menu labeling is an intervention for which efficacy depends on other factors, such as the simultaneous availability of attractive, highly palatable items [14]. External cues and emotional and physiological drivers often override rational thought when it comes to food consumption. This is consistent with the findings of previous studies [15]. However, fruit and vegetables were also highly regarded. The results indicate a need to increase concern about nutrition, as factors such as taste and appearance still appear to be more important considerations for most students.

The majority of the students interviewed believed the availability of calorie information enabled them to make healthier, more informed purchase decisions and “not grab junk food.” The positive feedback given by the interviewees corresponds with the significant decrease in gross calories and fat purchased per student recorded when calorie labels were present. The students believed the information helped them to estimate the calories they consumed at lunch and to choose smaller portion sizes. This finding is consistent with research by Girz et al. [16], who noted a decrease in portion size amongst a sample of adults when menu labels were introduced. These findings demonstrate that modifying the school environment by introducing point-of-purchase calorie labels led to a positive behavior change in students. In the current study, interviewed adolescents appeared to have a lack of awareness or to have forgotten about the importance of calorie content of food. The success of the calorie labeling may be attributed to the labels presenting a daily reminder of this information. The decrease in calories observed in this study may be partly attributed to the nutrition education that is integrated into the school curriculum. A recent meta-analysis found that when calorie labels were provided along with contextual or interpretive nutritional information, consumers selected fewer calories [17]. Interestingly, despite the decrease in gross calories purchased by students when calorie labels were present, most interviewees did not admit to discussing the labels with their peers. It has been identified in the literature that students think it is important for social reasons not to be overweight and can struggle with the pressure to be a socially desirable body weight [18]. It could be hypothesized there is some stigma attached to discussing nutritionally related topics as one student exclaimed: “we don't talk about that kind of stuff.” However, the support of friends for healthy eating has been positively associated with an increase in vegetable consumption [19]. Our results indicate there are barriers when it comes to adolescents discussing nutrition with their peers. The school should encourage conversation in the area of healthy eating and nutrition to rid any stigma these topics may attract.

### 4.1. Strengths and Limitations of the Study

The present study has a number of strengths. The majority of studies previously conducted have examined the effects of calorie posting in fast-food or chain-style restaurants but school cafeterias have largely been neglected. The current study contributes to the limited research in this area as we analyzed the impact of calorie labeling on a school cafeteria and the students reactions to these labels. The study also measured adolescents' gross consumption rather than their behavioral intent. Consequently, social desirability bias in reporting is of less concern and internal validity is probably better than studies that only measured behavioral intent [20–22]. Participants were also exposed to the calorie information for a long period of time (17 days) in a familiar setting, whereas simulation studies of intended or hypothetical food choices fail to incorporate the social nature of food choices. The study also included both sexes, although these were not examined separately.

Weight gain can occur over time from relatively small differences between the number of calories consumed and calories expended (e.g., 50 to 100 calories per day) [4]. Conversely, calorie reductions as small as those seen in the present study may be enough to lead to sustained weight loss over time and may be more realistic than dramatic changes.

The study also has several limitations. First, the results only demonstrate the positive short-term benefits of calorie labeling and it is not possible to conclude that these results will be stable for long term due to a lack of follow-up study. Second, only one middle school, in one school district, was examined so it is unclear how demographic variables may influence responses to calorie information in other settings. Furthermore, previous studies have demonstrated that adult males tend to choose higher calorie meals than adult females. The current study design did not allow for separation by sex and therefore, we cannot ascertain if the findings were strongly influenced by just one sex [23]. Third, it was not possible to explore whether the effects of calorie information will lead to long-term weight loss or weight maintenance in the adolescent population. Moreover, our findings could have been influenced by external factors such as weather differences or physical activity expenditure differences but as we conducted our study over the winter period on the same number of contact days we have minimized this influence to the extent possible. It is also possible that children consumed fewer calories at school during the intervention but compensated for these calories later in the day; we have not measured this aspect. We also acknowledge that we assessed gross calories based on food selection rather than food intake and therefore we can only state that children selected fewer calories. These findings provide preliminary insight into middle school student behavior. Additional research in different settings with different age groups is warranted. For example, Yamamoto et al. [20] demonstrated that calorie information influenced reported purchase intentions at some food outlets but not others. These results suggest that our findings may apply to some school settings but not all. In any case the current study contributes to the limited literature available in the area of calorie labeling in school settings.

## 5. Conclusions

No single solution will reverse the adolescent obesity epidemic. Calorie labels in schools are no exception but they may be part of the solution. As part of a broader movement, calorie labeling may induce systemic effects that over time could initiate a virtuous cycle. For instance, publishing caloric data at the point-of-purchase in schools could increase awareness and change student purchasing decisions outside of the school setting, leading to fewer calories consumed. The results of this CBPR study are encouraging and indicate that calorie labeling has a beneficial effect on student food choices. Calorie labels at the point-of-purchase are a promising approach to tackling the growing problem of adolescent obesity.

## Figures and Tables

**Table 1:** Changes in calorie and fat consumption.

Meal	Kcal consumed Per student		Grams fat consumed per student	
	Prelabel	Postlabel	Prelabel	Postlabel
1	740	766	26.0	27.2
2	725	602	28.3	23.5
3	596	576	16.3	16.2
4	807	748	34.0	30.9
6	819	672	24.6	20.8
7	669	662	27.6	26.9
8	570	545	17.1	16.2
9	430	441	11.7	13.1
10	745	767	27.9	28.5
11	711	617	23.5	20.0
12	617	579	19.7	17.0
13	773	745	33.4	31.4
14	689	641	25.1	22.5
15	559	467	17.8	13.4
16	868	737	23.6	17.2
17	366	363	13.4	12.1
Average	**668 ± 138**	**621 ± 122**	**23.1 ± 6.6**	**21.1 ± 6.4**

Kcal/student			Total Fat g/student	
Mean difference = −47			Mean difference = −2.1	
Std. Dev of difference = 14			Std. Dev of difference = 0.6	
95% CI = −77 to −18			95% CI = −3.3 to −0.9	
*P* = 0.0040			*P* = 0.0025	

^*^No consumption data were reported for any entrees on 2/8/2010. This menu served on dates 1/11/2010 and 2/8/2010 was excluded from the analysis (day 5).

**Table 2:** Sex and grade of students participating in menu labeling interviews (*n* = 32).

	Males	Females	Total
Sixth grade	6	6	12
Seventh grade	4	7	11
Eighth grade	5	4	9
Total	**15**	**17**	**32**

**Table 3:** Five significant themes.

Themes	Quotations
Theme 1: Students want nutrition information and felt it was a school's duty to help them achieve and maintain a healthy weight.	
Students want schools to provide nutrition information to help them make healthier food choices and achieve a healthy weight. Students wanted the school to (i) provide the calorie labels at point of purchase, (ii) provide more opportunities for activity, (iii) discuss nutrition more in the classroom (iv) only give healthy choices at lunch, (v) announce what the healthy cafeteria options are everyday	“They 're {labels} good and I think they should start showing them more often” “I think they should put those little signs back up” More “variety of fruit and vegetables” “selections of healthy food”, “food with less fat” and “with lower calories” “Give extra time at recess. Cause they talked about an hour of exercise that you need every day in health class” “offer sweet things less frequently”
Students recognized that the school was trying to take measures to combat obesity.	“with labels they're {school}, at least, attempting to do something about it” “My teacher mentioned the labels once and that's when I went looking for them”
Many students want classroom based nutritional education alongside menu labeling in the cafeteria.	“I think they should have an assembly and talk to everybody about labels”

Theme 2: Student understanding of nutrition and use of nutritional information were related to home environment.	
Students with parents who discussed nutrition at home and used nutritional labels were more likely to (i) notice and use the lunchroom labels, (ii) use nutritional labels on prepackaged food, (iii) have a greater overall awareness about nutrition and healthy eating, (iv) place greater importance on nutrition in general	“Sometimes my dad just tells me we gotta eat healthy” “she {mother} likes it when things have labels so that she can make a choice” “I'd go home and talk to my parents and tell them what we had at school and we would talk about it” “We look up to see how much sugar it has for fun. We just start guessing, then someone gets it right” “My mom, my sisters and me. We look at the nutrition label thing”
Students who did not discuss nutrition at home (i) had poor nutritional knowledge, (ii) were less likely to use the calorie labels, (iii) were to assess the schools obesity problem	Q. “how is it that you make decisions about what you're gonna eat?” A. “My dad, he usually just says he eats what he wants” “We just, kind of, eat whatever there is” “We don't talk about nutrition” Q. “Do you think there's a lot or a little problem with overweight at the school?”… A. “Not that much.”
Some students used their knowledge of the school calorie labels back in their home.	“I use it, like, when I prepare some foods at home”

Theme 3: Taste preference, nutrition, and being a healthy weight are important to most of the students.	
Taste, nutrition, and appearance are the most important factors for students when choosing food. Taste was the most significant factor for students Nutritious food needs to taste and look good in order for the students to eat it. Some students highly prioritized taste over nutritional content of food	“Taste and portion size and to see how healthy it is for me. Those are the 3 most important things.” “If it don't taste and look good, I'm not gonna eat it” “I don't really care how high the calories are as long as it tastes good”

Theme 4: Most but not all students admitted to noticing and using the calorie labels to make healthier food choices.	
The students who claimed to use the labels all used them to make healthier lower calorie food choices. Reasons given for menu labeling use include (i) helping to make lower calorie food choices, (ii) helping when comparing the calorie content of food, (iii) being easier to make healthier food choices, (iv) giving an idea of calories consumed at lunch each day, (v) helping when choosing smaller potions, (vi) reminding them to think of nutrition when choosing a food	“now I get something that doesn't have as much calories” “I didn't get the stuff I used to get…I ate more fruit” “I tried to grab low calorie, healthy foods and not grab junk food” “I had a piece of paper with me the second day to see how many calories I had during lunch” “I took a smaller plate of chips” “helped me choose the right food to eat” “It helps you think about nutrition”

Theme 5: The calorie labels and nutritionally related topics in general were not discussed among students.	
Most students did not discuss the presence of the labels in the cafeteria with their peers. Initial interest shown by students prompted some discussion.	“I have never heard a classmate of mine say anything” “At first everyone said what's up with the food calories” “We did talk about it for a little bit, but then we would just focus on the food”
